# Hospital admission of older patients with mild traumatic brain injury and traumatic intracranial hemorrhage: is it always necessary?

**DOI:** 10.1007/s00068-024-02671-z

**Published:** 2025-01-12

**Authors:** Juliette A. L. Santing, Maxime Van Gent, Crispijn L. Van Den Brand, Joukje Van Der Naalt, Korné Jellema

**Affiliations:** 1 Department of Neurology, Haaglanden Medical Center, PO Box 432, 2501 CK The Hague, The Netherlands; 2 Department of Neurology, Alrijne Hospital, PO Box 4220, 2350 CC Leiderdorp, The Netherlands; 3 Department of Emergency Medicine, Erasmus Medical Center, PO Box 2040, 3000 CA Rotterdam, The Netherlands; 4 Department of Emergency Medicine, Haaglanden Medical Center, PO Box 432, 2501 CK The Hague, The Netherlands; 5 Department of Neurology, University of Groningen, University Medical Center Groningen, PO Box 30.001, 9700 RB Groningen, The Netherlands

**Keywords:** Mild traumatic brain injury, Traumatic intracranial hemorrhage, Older adults, Hospital admission

## Abstract

**Background and importance:**

Traumatic intracranial hemorrhage (tICH) after mild traumatic brain injury (mTBI) is not uncommon in the elderly. Often, these patients are admitted to the hospital for observation. The necessity of admission in the absence of clinically important intracranial injuries is however unclear.

**Objectives:**

The objective of this study is to identify which factors additional to tICH affect the risk of this outcome and to evaluate the differences in the risk of adverse outcome in younger and older mTBI patients with tICH.

**Design, setting, and participants:**

This retrospective study assessed adult (≥ 16 years) mTBI patients with tICH admitted to a Level 1 trauma center between January 2017 and October 2020.

**Outcome measures and analysis:**

Patients were stratified into two groups, age < 65 years and age ≥ 65 years. Adverse outcome due to tICH was assessed using a composite adverse outcome which comprised either, a drop in GCS by more than 1 point, progression of or new neurological deficits, seizure activity, progression of tICH on repeated neuroimaging after clinical deterioration, a neurosurgical intervention, a readmission within three months of injury related to TBI, or death. Logistic regression analysis was used to identify independent predictors of the composite adverse outcome.

**Main results:**

In total, 332 mTBI patients with tICH were enrolled in our study. Older mTBI patients with tICH met the criteria for the composite adverse outcome significantly more often than younger patients (12.6% 95% CI 8.0–17.0% vs. 4.9%, 95% CI 1.0–9.0%, p = 0.033). The univariate analysis showed that a neurological deficit (OR 6.55, 95% CI 2.37–18.08) or a SDH on admission (OR 3.13, 95% CI 1.40–6.99) was positively associated with the composite adverse outcome in older patients. The presence of isolated traumatic SAH (tSAH) was associated with a decreased risk of the composite adverse outcome (OR 0.10, 95% CI 0.01–0.71). Multivariate analysis was not possible.

**Conclusion:**

Serious adverse outcomes are frequently observed in older mTBI patients with tICH. Nonetheless, our findings suggest that older patients with an isolated tSAH are at low-risk for deterioration and may be directly discharged from the ED after a short period of observation.

**Supplementary Information:**

The online version contains supplementary material available at 10.1007/s00068-024-02671-z.

## Introduction

Mild traumatic brain injury (mTBI) is one of the most common acute neurological disorders with the highest incidence in older adults (defined as ≥ 65 years old) in many countries worldwide [1]. Approximately 10% of patients with mTBI will have a traumatic intracranial hemorrhage (tICH), albeit this risk is higher in older age groups, with an incidence up to 18% in patients over the age of 70 years [2]. Although older age is associated with poorer outcome following mTBI compared to younger patients, it is important to realize that most mTBI patients do not have life-threatening tICH or require a neurosurgical intervention, and still return to preinjury functioning [3, 4].

Current guidelines on mTBI advise hospital admission of all patients with tICH because of the potential risk of hematoma progression and deterioration [5–8]. The clinical necessity of admission in the absence of clinically important intracranial injuries and other factors that would warrant a hospital admission (for example a GCS score that has not returned to 15, comorbidities, frailty) [5–8] is however unclear [9], including fully conscious patients using antithrombotics. There is further no consensus concerning the optimal inpatient care of the low-risk mTBI patients with tICH. Management varies from ICU admission, standard consultation of a neurosurgeon, and repeating head computed tomography (CT), to discharge home after a period of observation in the Emergency Department (ED) [10–19].

Since mTBI-related ED visits and hospital admissions are rapidly growing, particularly among the elderly [20], there is an increasing demand to improve the efficacy of the management strategy in this population. Moreover, studies have demonstrated that older patients have an increased risk of functional decline due to hospitalization and are more susceptible to hospital-acquired complications, such as delirium, falls, and infections [21–23]. To avoid unnecessary and potentially harmful hospital admissions, it would be beneficial to identify a subset of older mTBI patients with tICH who are at low risk for deterioration and could be discharged home from the ED after a short period of observation.

Therefore, the primary aim of this study was to identify which factors in addition to tICH affect the risk of adverse outcome after mTBI with tICH. The identification of such risk factors may facilitate the safe discharge of patients without these risks from the ED. The secondary aim was to evaluate the difference in the risk of adverse outcome in older mTBI patients compared to younger patients with tICH to identify specific age-related factors.

## Methods

### Study design and setting

We conducted a single center, observational cohort study at the three EDs of Haaglanden Medical Center from January 2017 to October 2020 comprising one Level I trauma center and two Level II trauma centers, all located in an urban setting. Consecutive patients aged 16 years and older who visited the ED within 24 h after mTBI with radiological evidence of acute tICH on initial head CT, admitted to the hospital were included. Mild TBI was defined as presenting with a GCS score of 13–15 and if present; posttraumatic amnesia (PTA) of ≤ 24 h and/or loss of consciousness (LOC) of ≤ 30 min. Radiographic evidence of tICH included isolated traumatic subarachnoid hemorrhage (tSAH), extradural hemorrhage (EDH), subdural hemorrhage (SDH) with an acute component, intraventricular hemorrhage (IVH), intraparenchymal hemorrhage/contusion, diffuse axonal injury, or a combination on cranial CT-scan as interpreted by the faculty neuroradiologist. Patients were excluded if the ICH was of non-traumatic nature, in case of discharge from the ED, or a history of previous mTBI with tICH. The study was approved by the local ethical review board.

### Data collection

Patients were categorized into two age groups based on their age at the time of injury into older mTBI (age ≥ 65 years) and young mTBI (age < 65 years) patients. Patients were identified by examination of the ED medical electronic registry with a main complaint of International Classification of Diseases, Tenth Revision diagnosis relating to TBI. Demographic characteristics, trauma mechanisms, and risk factors for intracranial complications used in the Canadian CT head rule (CCHR), the New Orleans criteria (NOC), National Institute for Health and Care Excellence (NICE), and CT in head injury patients (CHIP) rule [24] were extracted from data recorded at the time of presentation at the ED. The electronic patient files were reviewed for evidence of clinical deterioration, interventions, or death due to TBI. We assessed the electronic health records after discharge for evidence of deterioration, readmission, intervention, or death in the 3 months following the initial ED attendance. JS and MG performed the data extraction.

### Outcome measures

The outcomes of interest were measures indicating a need for hospital admission previously used in the literature [25]: death due to TBI, neurosurgical intervention for TBI or any other measure of clinical deterioration within 3 months. We defined clinical deterioration as a decrease in consciousness by more than 1 point (GCS); progression of or new neurological deficits; seizure activity; progression of tICH on repeated neuroimaging after clinical deterioration, and readmission related to TBI, all within three months of the initial ED attendance. Finally, a composite adverse outcome including death, neurosurgical intervention, or any other measure of clinical deterioration due to TBI within three months of injury was determined. When reason for death was unknown, it was attributed to TBI deterioration.

### Statistical analysis

Statistical analysis was performed using IBM SPSS Statistics versions 26 and 27. Categorical data are described as numbers and percentages, and continuous data with median values with interquartile ranges (IQR). Comparisons of demographic and clinical parameters between patients younger and equal or older than 65 years of age were conducted using the Fisher's exact test by doubling the exact one-tailed probability for categorical data. Outcomes per type of ICH and age group were provided with descriptive statistics. Additionally, we executed a univariate logistic regression analysis for the identification of risk factors associated with the composite adverse outcome. Variables that were significantly associated with the composite adverse outcome (p < 0.05) were included in multivariate regression analysis, if there were enough events per variable (n = 10 events [for the composite adverse outcome] per independent variable) to perform the analysis. These results were presented with an odds ratio (OR) and corresponding 95% CI.

## Results

### Patient characteristics

In total, 332 mTBI patients with tICH were included during the study period. Of these, 102 patients (30.7%) were classified as young and 230 patients (69.3%) were classified as older mTBI patients. Table 1 shows the patients characteristics per age group. Compared to younger patients, older patient’s were more often female, used more often antithrombotics, experienced more frequent a fall from standing as trauma mechanism, had less often posttraumatic symptoms, had more often an initial GCS of 15, and were more often diagnosed with SDH as type of hemorrhage.

**Table 1:** Baseline demographic and clinical characteristics per age group

Characteristics	Patients < 65 years (n = 102)	Patients ≥ 65 years (n = 230)	p-value
Age (years), median (IQR)	52 (36.50–59.25)	79 (73.75–86.25)	–
Men, n (%)	62/102 (60.8)	109/230 (47.4)	0.036
Antithrombotic use, n (%)			
None	83/102 (81.4)	78/230 (33.9)	< 0.001
VKA	4/102 (3.9)	38/230 (16.5)	0.001
DOAC	2/102 (2.0)	23/230 (10.0)	0.010
APT	11/102 (10.8)	70/230 (30.4)	< 0.001
DAPT	1/102 (1.0)	15/230 (6.5)	0.030
Combination	1/102 (1.0)	6/230 (2.6)	0.623
Reversal of anticoagulants, n (%)	3/7 (42.9)	46/67 (68.7)	0.339
Mechanism of injury, n (%)			
Fall from standing	25/98 (25.5)	139/207 (67.1)	< 0.001
Fall from height	11/98 (11.2)	32/207 (15.5)	0.552
Fall from bicycle	29/98 (29.6)	21/207 (10.1)	< 0.001
Pedestrian or cyclist vs. vehicle	14/98 (14.3)	8/207 (3.9)	0.003
Other RTA	11/98 (11.2)	12/207 (5.8)	0.154
Bump head against object	1/98 (1.0)	2/207 (1.0)	1.000
Abuse	7/98 (7.1)	3/207 (1.4)	0.028
GCS, n (%)			
13	11/102 (10.8)	10/230 (4.3)	0.054
14	27/102 (26.5)	51/230 (22.2)	0.474
15	64/102 (62.7)	169/230 (73.5)	0.067
Posttraumatic symptoms, n (%)			
LOC	48/77 (62.3)	71/187 (38.0)	< 0.001
PTA	47/77 (61.0)	80/200 (40.0)	0.002
Anisocoria	2/102 (2.0)	1/229 (0.4)	0.460
Vomiting	23/88 (26.1)	22/212 (10.3)	0.001
Neurological deficit	6/102 (5.9)	19/229 (8.3)	0.600
Posttraumatic seizure	4/90 (4.4)	1/229 (0.4)	0.047
Visible injury to the head	61/99 (61.6)	160/227 (70.5)	0.150
Signs of skull fracture	25/99 (25.2)	20/226 (8.8)	< 0.001
Type of hemorrhage, n (%)			
Subarachnoid hemorrhage	27/102 (26.5)	56/230 (24.3)	0.680
Contusion	14/102 (13.7)	27/230 (11.7)	0.732
Subdural	19/102 (18.6)	87/230 (37.8)	< 0.001
Multiple lesions	41/102 (40.2)	58/230 (25.2)	0.001
Others*	1/102 (1.0)	2/230 (0.8)	1.000
Skull fracture, n (%)	35/102 (34.3)	23/230 (10.0)	< 0.001

*APT* antiplatelet therapy, *DAPT* double antiplatelet therapy, *DOAC* direct oral anticoagulant, *GCS* Glasgow Coma Scale, *IQR* interquartile range, *LOC* loss of consciousness, *PTA* posttraumatic amnesia, *RTA* road traffic accident, *VKA* vitamin K antagonist

### Outcomes

Thirty-four patients (10.2% 95% CI 7.2–14.0%) met the criteria for the composite adverse outcome. Thirty patients (9.0%, 95% CI 6.5–12.6%) experienced clinical deterioration, 17 patients (5.1%, 95% CI 3.0–8.1%) needed a neurosurgical intervention and 9 patients (2.7%, 95% CI 1.2–5.1%) died due to tICH. Older mTBI patients met the criteria for the composite adverse outcome significantly more often than younger mTBI patients (12.6%; 95% CI 8.0–17.0% vs. 4.9%; 95% CI 1.0–9.0%, p = 0.033). Table 2 shows the individual components of the composite adverse outcome per age group. The outcomes of interest for each type of ICH and age group is shown in Table 3. Particularly patients with a SDH or multiple intracranial traumatic lesions met the criteria for the composite adverse outcome.

**Table 2:** Risk of deterioration per age group

Outcome, n (%)	Patients < 65 years (n = 102)	Patients ≥ 65 years (n = 230)	p-value
Clinical deterioration	5 (4.9)	25 (10.9)	0.080
GCS deterioration	2 (2.0)	15 (6.5)	0.082
New or progressive neurological deficit	2 (2.0)	15 (6.5)	0.561
Seizure	1 (1.0)	4 (1.7)	0.601
Progression of ICH on neuroimaging	5 (4.9)	23 (10.0)	0.123
Neurosurgical intervention	3 (2.9)	14 (6.1)	0.230
Death	1 (1.0)	7 (3.0)	0.196
Composite outcome*	5 (4.9)	29 (12.6)	0.033

*GCS* Glasgow Coma Scale, *ICH* intracranial hemorrhage

*The individual components of the composite outcome (clinical deterioration, neurosurgical intervention, or death) do not add up to the composite oucome, as patients can have more than one individual component

**Table 3:** Outcomes per type of ICH and age group

Subgroup	No. (%) of patients									
	Age < 65 years					Age ≥ 65 years				
Characteristic	SAH n = 27	Contusion n = 14	SDH n = 19	Multiple lesions n = 41	Others n = 1	SAH n = 56	Contusion n = 27	SDH n = 87	Multiple lesions n = 58	Others n = 2
Outcome										
Clinical deterioration	0 (0)	0 (0)	2 (10.5)	3 (7.3)	0 (0)	1 (1.7)	1 (3.7)	15 (17.2)*	8 (13.8)	0 (0)
Neurosurgical intervention	0 (0)	0 (0)	2 (10.5)	1 (2.4)	0 (0)	0 (0)	1 (3.7)	11 (12.6)**	2 (3.4)	0 (0)
Death	0 (0)	0 (0)	0 (0)	1 (2.4)	0 (0)	0 (0)	0 (0)	4 (4.6)***	3 (5.2)	0 (0)
Composite outcome	0 (0)	0 (0)	2 (10.5)	3 (7.3)	0 (0)	1 (1.7)	1 (3.7)	19 (21.8)	8 (13.8)	0 (0)

*EDH* extradural hemorrhage, *ICH* intracranial hemorrhage, *IVH* intraventricular hemorrhage, *N/A* not applicable, *SAH* subarachnoid hemorrhage, *SDH* subdural hemorrhage

*In 8 patients related to chronic SDH development

**In 4 patients related to chronic SDH development and in 2 patients related to a CSF leak

***In 2 patients related to chronic SDH development

Presence of neurological deficits was associated with the composite adverse outcome in both young (OR 47.00, 95% CI 5.60–79.17) and older mTBI patients (OR 6.55, 95% CI 2.37–18.08) (using univariate analysis), as well as a SDH in older mTBI patients (OR 3.13, 95% CI 1.40–6.99). Isolated tSAH as type of hemorrhage was associated with a decreased risk of the composite adverse outcome in older mTBI patients (OR 0.10, 95% CI 0.01–0.71) (Table 4). Multi-variate analyses could not be performed because of a low number of events per variable.

**Table 4:** Univariable analysis for factors associated with the combined endpoint

Subgroup	Patients < 65 years (n = 102)			Patients ≥ 65 years (n = 230)		
Variable	OR	95% CI	P-value	OR	95% CI	p-value
Female sex	0.99	0.16–6.21	0.99	0.70	0.32–1.53	0.37
Antithrombotic use						
None	0.91	0.10–8.65	0.93	0.71	0.30–1.69	0.44
VKA	0.00	–	1.00	0.79	0.26–2.40	0.67
DOAC	0.00	–	1.00	1.53	0.48–4.87	0.47
APT	2.18	0.22–21.41	0.51	1.03	0.45–2.40	0.94
DAPT	0.0	–	1.00	2.76	0.82–9.34	0.10
Combination	0.00	–	1.00	0.00	–	1.00
Reversal of anticoagulants	0.00	–	1.00	0.91	0.35–2.40	0.85
Trauma mechanism						
Fall from standing	0.76	0.08–7.14	0.79	1.11	0.50–2.46	0.81
Fall from height	0.00	–	1.00	0.66	0.19–2.31	0.51
Fall from bicycle	1.79	0.27–10.92	0.56	0.32	0.04–2.50	0.28
Pedestrian or cyclist vs. vehicle	1.62	0.17–15.60	0.69	0.99	0.12–8.35	0.99
RTA	0.00	–	1.00	0.62	0.08–4.96	0.65
Bump head against object	0.00	–	1.00	0.00	–	1.00
Abuse	0.00	–	1.00	0.00	–	1.00
GCS						
15	2.47	0.27–22.92	0.46	0.65	0.28–1.48	0.30
14	0.68	0.07–6.39	0.74	0.46	0.58–3.38	0.46
13	0.00	–	1.00	1.79	0.36–8.86	0.48
Posttraumatic symptoms						
LOC	1.22	0.11–14.05	0.88	0.61	0.27–1.66	0.33
PTA	2.70	0.29–25.37	0.39	0.79	0.30–2.07	0.63
Anisocoria	0.00	–	1.0	0.00	–	1.00
Vomiting	0.00	–	1.0	2.65	0.88–7.98	0.08
Neurological deficit	47.00	5.60–79.17	< 0.001*	6.55	2.37–18.08	< 0.001*
Posttraumatic seizure	0.00	–	1.0	0.00	–	1.00
Visible injury	0.38	0.06–2.37	0.3	0.94	0.39–2.27	0.88
Signs of skull fracture	0.99	0.10–9.93	1.0	1.40	0.38–5.16	0.61
Type of ICH						
SAH	0.00	–	1.00	0.10	0.01–0.71	0.02*
Contusion	0.000	–	1.00	0.24	0.03–1.84	0.17
SDH	3.14	0.49–20.24	0.23	3.13	1.40–6.99	0.01*
Multiple lesions	2.33	0.37–14.59	0.37	1.40	0.60–3.27	0.44
Others	0.00	–	1.00	0.00	–	1.00
Skull fracture	3.05	0.49–19.16	0.24	0.63	0.14–2.85	0.55

*APT* antiplatelet therapy, *CI* confidence interval, *DOAC* direct oral anticoagulant, *DAPT* dual antiplatelet therapy, *EDH* extradural hemorrhage, *GCS* Glasgow Coma Scale, *ICH* intracranial hemorrhage, *IVH* intraventricular hemorrhage, *LOC* loss of consciousness, *OR* odds ratio, *PTA* post-traumatic amnesia, *SAH* subarachnoid hemorrhage, *SDH* subdural hemorrhage, *VKA* vitamin K antagonist, *RTA* road traffic accident

In our cohort, one patient with a tSAH met the criteria for the composite adverse outcome. This 73-year-old male was readmitted 5 days after trauma due to persistent headache and slight progression of SAH on a repeated head CT. Following a brief period of observation with adequate pain management, he could be discharged home. None of the patients with tSAH experienced any other measure of clinical deterioration, needed a neurosurgical intervention, or died. Details of all the patients who met the criteria for the composite adverse outcome sorted by type of ICH are provided in Supplementary Table 1.

## Discussion

In our cohort of 332 mTBI patients with tICH, we found that one in ten patients showed the composite adverse outcome indicating adverse outcome with clinically relevant deterioration, neurosurgical intervention, or death. Older patients had the highest prevalence of adverse outcomes (12.6%). In older patients, the presence of neurological deficits, or a SDH upon presentation was associated with a six and three times higher risk of the composite adverse outcome respectively, whereas the presence of an isolated tSAH was associated with a lower risk (OR 0.1).

Outcomes in mTBI patients with tICH vary widely. Similar to our findings, a meta-analysis [24] estimated a pooled risk of clinical deterioration of 11.7%, risk of neurosurgery of 3.5%, and risk of death of 1.4%. However, the median age of patients in all the included studies was lower than 60 years. When mTBI patients were partitioned at 65 years of age in the current study, we observed that these figures were higher in older patients (clinical deterioration: 10.9% vs. 4.9%, neurosurgical intervention: 6.1% vs. 2.9%, and death: 3.5% vs. 1.0% respectively). Several studies have identified older age as risk factor for adverse outcomes [25–30]. Preexisting clinical conditions are associated with even worse outcomes post-TBI, and may be more predictive of admission and outcome after mTBI than injury characteristics in older adults [1]. Nonetheless, there is also a substantial number of older patients with mTBI and tICH who recover well and uneventfully, suggesting a possibility to identify a subgroup of patients who may possibly be safely discharged from the ED after a period of observation when no other factors that would warrant a hospital admission are present.

In our study, having a neurological deficit was associated with adverse outcome in elderly mTBI patients with tICH. Presence of neurological deficits could be interpreted as an indicator of more severe intracranial injury resulting in less favorable outcome. This is supported by results of recent large, multi-center observational studies reporting a significant association between more severe CT abnormalities and poor outcome in adult mTBI patients with an additive effect of older age [31–33].

Regarding the type of tICH, we found in our study that the presence of a SDH was associated with adverse outcome in older mTBI patients. In general, subdural hemorrhages after mTBI are more often seen in older patients due to various reasons, such as cerebral atrophy leading to strain on bridging cortical veins, more fall-related TBIs with focal impact, comorbidities and polypharmacy facilitating SDH formation and expansion [1]. Prior studies in mTBI patients also found that SDHs were associated with worse outcomes. A retrospective cohort study reported that more than 40% of patients with a SDH volume of ≥ 10 cc experienced a change in neurological status that resulted in higher level of care, prolonged hospitalization or a neurosurgical procedure [34]. In addition, it has been reported that mTBI patients with a SDH are more likely to require a neurosurgical intervention compared to other types of tICH [9, 35]. These findings suggest that all mTBI patients with a SDH, especially those aged above 65 years or with a large SDH (≥ 10 cc), could benefit from an inpatient admission for neurological observation. It is worth noticing that almost 10% of the mTBI patients with a SDH in our study deteriorated after a hospital admission for at least 24 h and discharge in normal neurological condition, most often due to progression of initially non-operated acute SDH to chronic SDH. The data therefore do not speak to the safety of an ED discharge protocol for mTBI and associated tICH. This finding underlines the importance of patient instructions upon discharge from the hospital and might suggest the need for close follow-up assessment in the first months.

Unlike older mTBI patients with a SDH, we found that older mTBI patients with an isolated SAH had a significant lower risk of an unfavorable outcome. This observation is in accordance with other results seen in younger mTBI adults with an isolated SAH. In a single center retrospective study, none of the 371 patients with mTBI and tSAH who were admitted to an ED observation experienced neurologic deterioration and all were discharged uneventfully [13]. A meta-analysis of over 15,000 mTBI patients with isolated tSAH patients also showed that this subgroup of mTBI patients with tICH experience very low rates of radiological progression and neurologic deterioration, and rarely require a neurosurgical intervention or die due to brain injury [36]. In this study cohort, mTBI patients with tSAH represented 25% of all hospital admissions, suggesting a potential to manage a substantial part of mTBI patients with tICH in the community. Indeed, there are more indications for a hospital admission than tICH alone, such as a GCS score that has not returned to 15, persistent PTA, or other injuries. However, even if half of tSAH patients could be discharged home, this would result in more discharges than numbers of 5 to 10% found in previous studies [37, 38]. A prospective study is needed to investigate the clinical and economic impact of discharging mTBI patients with tSAH directly from the ED.

Currently, some consensus-derived risk tools are developed and used that might aid in distinguishing the low-risk mTBI patients with tICH suitable for discharge [37, 38]. These models however lack specificity and are not validated in adequately powered prospective cohorts and are not applicable to older patients, as older age and use of antithrombotics, which are frequently used by older adults, are considered as predictors of high-risk status. The development of a specific decision rule, that identifies low-risk older mTBI patients with tICH and mTBI patients with tICH who can be safely discharged from the ED, is required. This has the potential to diminish overcrowding in the hospital, save costs of expensive inpatient stays, and improve patient outcomes as well by minimizing the risk of hospital-acquired conditions.

If a strategy for discharge from the ED in a subset of low-risk older mTBI patients with tICH is possible, it is necessary to provide a structured follow-up pathway, because it cannot beforehand be assumed that these patients will fully recover to their preinjury functioning. Research shows that a substantial part of mTBI patients with few complaints early after injury, develop complaints at a later stage leading to less functional outcome and lower quality of life [4]. Further, older mTBI patients may experience a slower recovery pattern due to higher comorbid burden and polypharmacy [39]. Proper outpatient follow-up after mTBI with tICH in older patients is therefore essential, irrespective of hospital admission, to actively identify and address unmet needs to improve patient outcome after mTBI with tICH.

### Strengths and limitations

A strength of this study is that our study assessed a wide range of patient and clinical characteristics in relation to adverse outcome with a long follow-up of 3 months in contrast to other studies. The study has also some limitations in addition to its single-center and retrospective design. First, the sample size was relatively small and our study did not comprise sufficient patients to draw conclusions about less common clinical characteristics and traumatic ICH types in relation to outcomes, such as EDH. This also limited our ability to make comparisons between traumatic ICH groups and to perform a multivariable analysis to identify predictors of the composite adverse outcome. Second, it should be noted that any new mTBI experienced by the included patients was not recorded as a separate event. Consequently, the cause of deterioration remains unclear in some patients, which may have resulted in an overreporting of adverse events. Last, it is possible that some patients with deterioration in the community who were not presented to our hospital were missed. However, the Electronic Health Record system of our hospital is linked to the national register of deceased persons. Furthermore, general practitioners and paramedic services rarely refer or transfer neurotrauma patients to other hospitals than the hospital where they are earlier seen. Therefore, we believe that this had no significant consequences on our results.

## Conclusion

This study shows that after 3 months follow-up, serious adverse outcomes are frequently observed in older mTBI patients with tICH, especially in patients with neurological deficits or with a SDH. Nevertheless, older patients with an isolated tSAH are at low-risk for deterioration and may be directly discharged from the ED after a short period of observation. As mTBI-related ED visits and hospital admissions among the elderly are rapidly increasing and a hospital admission itself poses risks to older patients, direct ED discharge offers an opportunity to reduce healthcare costs and clinical workload and to avoid the risk of hospital-acquired conditions. Adequate follow-up of patients to determine a favorable recovery pattern remains necessary in these patients.

## Supplementary Information

Below is the link to the electronic supplementary material.

Supplementary file1 (DOCX 21 KB)

## Data Availability

No datasets were generated or analysed during the current study.
