# Supplementary material for: Hospital admission of older patients with mild traumatic brain injury and traumatic intracranial hemorrhage: is it always necessary?
Source: Eur J Trauma Emerg Surg. 2025 Jan 12;51(1):8. doi: 10.1007/s00068-024-02671-z (PMC11725537; doi:10.1007/s00068-024-02671-z)
Supplement: Supplementary file 1 — Supplementary file1 (DOCX 21 KB) [file 68_2024_2671_MOESM1_ESM.docx]

*Supplementary Table 1: Details of the patients who met the criteria for the composite outcome.*

| Patient | Mechanism of injury | Initial GCS score | Repeat CT finding | Neurosurgical intervention | Mortality | Classification deterioration | Comments |
| --- | --- | --- | --- | --- | --- | --- | --- |
| *Subarachnoid Hemorrhage* | |  |  |  |  |  |  |
| Male, 73 years | Unknown | 15 | Slight progression SAH | No | No | Clinical deterioration *(progression of ICH on neuroimaging)* | Readmitted 5 days after trauma with headache and slight progression of SAH on CT, treated conservatively |
| *Contusion* | |  |  |  |  |  |  |
| Male, 86 years | Pedestrian or cyclist vs. vehicle | 15 | New SDH | Yes, burr holes | No | Clinical deterioration *(progression of ICH on neuroimaging)*  Neurosurgical intervention | Remained dizzy for weeks and consequently fell repeatedly with new bilateral chronic SDH on CT wherefore readmission ad treatment with burr holes |
| *Subdural Hemorrhage* | |  |  |  |  |  |  |
| Female, 82 years | RTA | 15 | Progression of SDH | No | No | Clinical deterioration *(new neurological deficit, progression of ICH on neuroimaging)* | Readmitted 17 days after trauma with new paresis and progression of SDH on CT, treated conservatively |
| Male, 86 years | Fall from standing | 15 | Progression of SDH | Yes, burr hole | No | Clinical deterioration *(new neurological deficit, GCS deterioration, progression of ICH on neuroimaging)*  Neurosurgical intervention | Readmitted 5 days after trauma because of new fall with GCS deterioration (GCS 13), new paresis and progression of SDH on CT, treated with burr hole |
| Male, 87 years | Fall from standing | 15 | Progression of SDH, rebleed | Yes, craniotomy | No | Clinical deterioration *(new neurological deficit, GCS deterioration, progression of ICH on neuroimaging)*  Neurosurgical intervention | Readmitted 21 days after trauma because follow-up CT showed progression of SDH. Possibly fell the previous day. Increased headache, GCS deterioration (GCS 13) and new paresis 29 days after trauma with rebleed SDH on CT, treated with a craniotomy |
| Female, 78 years | Fall from standing | 15 | NA | Yes, burr hole | No | Neurosurgical intervention | During admission treated with a burr hole 4 days after trauma due to invalidating neurological deficits |
| Male, 81 years | Fall from standing | 13 | Slight progression of SDH or new bleeding | Yes, craniotomy | No | Clinical deterioration (GCS deterioration, progression of ICH on neuroimaging)  Neurosurgical intervention | GCS deterioration 1 hour after ED presentation (GCS 9), wherefore emergency craniotomy (no CT repeated).  Readmitted 11 days after trauma due to cortex irritation (transient phatic disorder and headache) with slight progression of tICH or new bleeding on CT, treated conservatively |
| Female, 83 years | Fall from standing | 15 | Progression SDH with herniation | No | Yes | Clinical deterioration *(new neurological deficit, GCS deterioration, progression of ICH on neuroimaging)*  Death | GCS deterioration (GCS 4) and anisocoria 2 hours after trauma with progression of SDH on CT and herniation. No intervention. Died the next day. |
| Male, 88 years | Fall from height | 14 | Progression SDH with mass effect | No | Yes | Clinical deterioration *(GCS deterioration, progression of ICH on neuroimaging)*  Death | Readmitted 18 days after trauma because of GCS deterioration (GCS 4) after new fall, with progression of SDH with mass effect on CT. No intervention. Died 3 days later. |
| Female, 87 years | Fall from standing | 15 | Progression SDH with mass effect | No | Yes | Clinical deterioration *(GCS deterioration, progression of ICH on neuroimaging)*  Death | Readmitted 11 weeks after trauma after being found in bed with GCS deterioration (GCS 5) and progression of SDH with mass effect on CT. No intervention. Died the same day |
| Female, 78 years | Fall from standing | 14 | Progression SDH | No | No | Clinical deterioration *(new neurological deficit, progression of ICH on neuroimaging)* | New phatic disorder during admission one day after trauma with progression of SDH on CT, treated conservatively |
| Female, 94 years | Fall from standing | 15 | Progression SDH | Yes, burr hole | No | Clinical deterioration *(GCS deterioration, progression of ICH on neuroimaging)*  Neurosurgical intervention | Follow-up CT showed progression of SDH 15 days after trauma (no complaints).  Readmitted 5 weeks after trauma due to GCS deterioration (GCS 13) with progression of SDH on CT, treated with burr hole |
| Male, 95 years | Fall from standing | 15 | Progression SDH with mass effect | No | Yes | Clinical deterioration *(GCS deterioration, progression of ICH on neuroimaging)*  Death | GCS deterioration (GCS 6) and vomiting 3 hours after presentation in ED with progression of SDH on CT. No intervention. Died the next day |
| Female, 71 years | Unknown | 15 | Progression hygroma | No | No | Clinical deterioration *(new neurological deficit, progression of ICH on neuroimaging)* | Readmitted 5 weeks after trauma because of three ED presentations in one week with transient neurological symptoms, with progression of hygroma on CT, treated conservatively |
| Male, 57 years | Fall from bicycle | 15 | Minimal progression of SDH and new SAH and contusion | Yes, ELD | No | Clinical deterioration *(new neurological deficit, progression of ICH on neuroimaging)*  Neurosurgical intervention | Eye movement disorder during first day of admission with minimal progression of traumatic SDH and new SAH and contusion on CT. ELD placement 7 days after trauma to treat CSF leak due to skull fracture |
| Male, 81 years | Fall from standing | 15 | NA | Yes, burr hole | No | Neurosurgical intervention | During admission treated with a burr hole 2 days after trauma due to invalidating neurological deficits |
| Male, 87 years | Unknown | 14 | Progression SDH with mass effect | Yes, burr hole | No | Clinical deterioration *(GCS deterioration, new neurological deficit, progression of ICH on neuroimaging)*  Neurosurgical intervention | Readmitted 15 days after trauma due to GCS deterioration (GCS 13) and balance disorder with progression of SDH and mass effect on CT, treated with burr hole |
| Male, 88 years | Fall from standing | 15 | Progression of SDH | Yes, craniotomy | No | Clinical deterioration *(GCS deterioration, new neurological deficit, seizure, progression of ICH on neuroimaging)*  Neurosurgical intervention | During admission seizure after 2 days. Readmission 15 days after trauma because of GCS deterioration (GCS 14), progression neurological deficits and progression of SDH and on CT (two new falls in meantime). Further GCS deterioration (GCS 8) and progression of SDH on CT 17 days after trauma, treated with craniotomy |
| Male, 82 years | Fall from standing | 15 | NA | Yes, burr hole | No | Neurosurgical intervention | During admission treated with a burr hole 2 days after trauma due to invalidating neurological deficits |
| Female, 71 years | Fall from bicycle | 14 | NA | Yes, ELD | No | Neurosurgical intervention | ELD placement 4 days after trauma to treat CSF leak due to skull fracture |
| Female, 74 years | Unknown | 14 | No progression of SDH | No | Yes, unrelated to the trauma | Clinical deterioration *(progression neurological deficit)* | Anisocoria and progression of neurological deficit during first day of admission with no progression of SDH on CT. Died of acute myeloid leukemia 12 days after trauma |
| Female, 58 years | Fall from standing | 15 | Slight progression of SDH | Yes, burr hole | No | Clinical deterioration *(progression of ICH on neuroimaging)*  Neurosurgical intervention | New vomiting during first day of admission with slight progression of SDH. Burr hole 4 days after trauma |
| Female, 79 years | Fall from standing | 15 | Progression of SDH | Yes, burr hole | No | Clinical deterioration *(new neurological deficit, progression of ICH on neuroimaging)*  Neurosurgical intervention | After discharge two ED presentations (day 2 and 5 after trauma) with transient neurological symptoms with no progression on CT. Readmission 12 days after trauma due to persistent neurological deficits and progression of SDH on CT, treated with burr hole |
| *Multiple lesions* | |  |  |  |  |  |  |
| Male, 87 years | RTA | 15 | Progression of contusion and edema causing mass effect | No | Yes | Clinical deterioration (G*CS deterioration, progression of ICH on neuroimaging)*  Death | During admission, GCS deterioration (GCS 9) two days after trauma with progression of contusion and edema causing mass effect. No intervention. Died the same day |
| Female, 72 years | Fall from standing | 15 | Progression of SDH, new SDH | Yes, burr hole and craniotomy | Yes | Clinical deterioration *(new neurological deficit, GCS deterioration, progression of ICH on neuroimaging)*  Neurosurgical intervention | Readmission 9 days after trauma with a new phatic disorder with progression of SDH on CT, treated conservatively  Readmission 18 days after trauma due to GCS deterioration (GCS 13) with progression of SDH treated with a burr hole  Readmission 29 days after trauma with a fever and GCS deterioration (GCS 14) with progression of SDH and suspected infection, treated with antibiotics and a burr hole. Anisocoria and GCS deterioration the same day due to a new SDH treated with a craniotomy. Died 5 days later |
| Male, 74 years | Unknown | 13 | Progression of contusion | No | Yes, cause of death unknown | Clinical deterioration *(new neurological deficit, GCS deterioration, seizure, progression of ICH on neuroimaging)*  Death | New paralysis during first day of admission with progression of contusion on CT. Focal seizure, GCS deterioration (GCS 11) during admission two days after trauma with increase of edema on CT. Died 5 weeks after trauma at home, cause of death unknown |
| 84, male | Fall from standing | 15 | Progression SDH | No | No | Clinical deterioration *(new neurological deficit, progression of ICH on neuroimaging)* | New aphasia, facial asymmetry and paresis at the ED, with spontaneous improvement  Readmission 12 days after trauma due to recurrence of neurological deficits with progression of SDH on CT, treated conservatively |
| 66, male | Fall from standing | 14 | Progression of SDH | Yes, craniotomy | No | Clinical deterioration *(progression neurological deficit, seizure, GCS deterioration, progression of ICH on neuroimaging)*  Neurosurgical intervention | Progression of neurological deficits, GCS deterioration (GCS 8) and a seizure at the ED with slight progression of SDH on CT, treated with a craniotomy  Transient neurological deficits at home 4 weeks after trauma |
| 70, male | Fall from standing | 15 | Progression of SDH | No | No | Clinical deterioration *(progression neurological deficit, progression of ICH on neuroimaging)* | Readmission due to new facial asymmetry and leg paresis 14 days after trauma with progression of SDH on CT, treated conservatively |
| 58, female | Fall from bicycle | 14 | Slight progression of SAH and SDH | No | No | Clinical deterioration *(new neurological deficits, progression of ICH on neuroimaging)* | ED presentation 6 days after trauma because of a peripheral facial palsy due to fracture of the temporal bone with slight progression of SAH and SDH on MRI, treated conservatively |
| 82, female | Fall from standing | 14 | NA | No | No | Clinical deterioration *(seizure)* | Several seizures during admission 4 days after trauma |
| 17, male | Pedestrian or cyclist vs. vehicle | 15 | Slight progression of contusion and SAH | No | No | Clinical deterioration *(GCS deterioration, progression of ICH on neuroimaging)* | Vomiting and GCS deterioration (GCS 13) during first day of admission with slight progression of contusion and SAH on CT, treated conservatively |
| 84, female | Fall from standing | 14 | Progression of SDH | No | Yes | Clinical deterioration *(GCS deterioration), progression of ICH on neuroimaging)*  Death | ED presentation 12 days after trauma due to GCS deterioration (GCS 3) with progression of SDH on CT. No admission or intervention (respecting family’s wishes). Died 30 days after trauma in a nursing home) |
| 63, male | Unknown | 15 | Progression of tICH (several lesions) | Yes, craniotomy | Yes | Clinical deterioration *(GCS deterioration, seizure, progression of ICH on neuroimaging)*  Neurosurgical intervention  Death | GCS deterioration (GCS 3) during the first day of admission with progression of tICH (several lesions), treated with a craniotomy. Had several seizures two days after trauma. Died eight days after trauma due to traumatic brain jury and respiratory insufficiency |

CSF = Cerebrospinal fluid; CT = computed tomography; ED = Emergency Department; EDH = extradural hemorrhage; ELD = external lumbar drain; GCS = Glasgow Coma Scale; ICH = intracranial hemorrhage; IVH = intraventricular hemorrhage; SAH = subarachnoid hemorrhage; SDH = subdural hemorrhage; RTA = road traffic accident.
